# The Effect of the Extrusion Method on Processing and Selected Properties of Poly(3-hydroxybutyric-co-3-hydroxyvaleric Acid)-Based Biocomposites with Flax and Hemp Fibers

**DOI:** 10.3390/polym14245370

**Published:** 2022-12-08

**Authors:** Grzegorz Janowski, Wiesław Frącz, Łukasz Bąk, Tomasz Trzepieciński

**Affiliations:** 1 Department of Materials Forming and Processing, Rzeszow University of Technology, 35-959 Rzeszow, Poland; 2 Department of Manufacturing Processes and Production Engineering, Rzeszow University of Technology, 35-959 Rzeszow, Poland

**Keywords:** flax fibers, hemp fibers, single-screw extrusion, twin-screw extrusion, PHBV, biocomposites

## Abstract

The paper presents a comparative analysis of two extrusion methods of biocomposites with a poly(3-hydroxybutyrate-co-3-hydroxyvalerate acid) (PHBV) matrix filled with flax and hemp fibers in terms of biopolymer production, its processing in the further injection process, and an evaluation of the mechanical and functional properties of the products. Biocomposites containing 15% by weight of the filler were produced using single- and twin-screw extruders. The biocomposites were then processed by injection molding and then, among other things, the pressures in the mold cavity during processing were analyzed. The produced samples were tested by means of the following tests: uniaxial tensile strength, hardness, and impact tensile strength. The biocomposite’s microstructure was also analyzed using scanning electron microscopy (SEM), as were the shrinkage and water absorption of the manufactured products. In addition, thermal gravimetric analysis (TGA) and differential scanning calorimetry (DSC) tests were performed. It was found that the extrusion method changed significantly the geometry of the filler fibers and the processing capabilities of the manufactured materials. Significant differences in the mechanical and functional properties of the obtained biocomposite products were also found. On their basis, the advantages and disadvantages of both extrusion methods were discussed. Most of the obtained properties of injection products indicate the choice of single-screw extrusion. The products were characterized by slightly better mechanical properties and lower processing shrinkage. In turn, composites obtained by the screw method were characterized by lower water absorption and lower viscosity of the composite during injection molding.

## 1. Introduction

New polymeric materials with increasingly improved properties and lower production costs are still being sought. The growing ecological awareness of societies leads to the search for environmentally friendly materials with such characteristics as biodegradability, natural origin, and renewability. The materials are becoming more and more important. This shapes the direction of production and an improvement of a new type of polymeric materials, from non-renewable, difficult to degrade, or completely non-biodegradable polymers to renewable ones of natural origin which are fully biodegradable [1,2,3]. The search for and development of this type of material often requires some compromises in terms of production costs in order to ensure appropriate properties. The development of composites based on green polymers, i.e., those that are both of natural origin and fully biodegradable, is still progressing. The scope of development of this type of materials sets new areas for the production of composites with better mechanical properties, striving to achieve thermal stability and obtain the lowest possible production costs in order to commercialize them as soon as possible.

Cellulose fibers as a filler in a polymer matrix are characterized by low production costs compared to synthetic fibers, and they also have appropriate strength properties that can be a great alternative to synthetic fillers [4,5]. Biocomposites produced with the use of a filler in the form of fibers of plant origin and green polymers can be one of the methods of reducing production costs and obtaining the desired properties while maintaining full biodegradation. In addition, their development can be a great factor for the development of agriculture and rural areas [6,7,8,9,10,11].

Green polymers utilize beneficial features such as an ability to produce from renewable raw materials and biodegradation, and are unique and most desirable polymer material in the current problem of waste management. One of the interesting materials of this type are polyhydroxyalkanoates (PHA), which are characterized by biocompatibility, non-toxicity, and the fact that they are subject to enzymatic degradation. The main polymers from the PHA group include poly(3-hydroxybutyric acid) (PHB), which is produced in cells of Ralstonia eutropha bacteria as a reserve material [12,13,14,15]. In order to improve the properties of this polymer, its copolymer, PHBV, is synthesized, which is characterized by better processing and mechanical properties in relation to PHB, which gives the possibility of its wider application in the production of plastic products. However, it should be mentioned that PHBV is currently characterized by a rather high price compared to the most popular polymers such as polyethylene, polypropylene, etc. Therefore, it is important to take steps to reduce the price and improve the properties of products made of biopolymer. The use of fibers of plant origin as a filler in the polymer matrix offers a possibility of solving this problem. Thanks to this, it is expected that the properties of the obtained moldings will be removed, and the price of the product reduced [16,17,18].

Positive features of cellulose fillers, such as low density at maintaining high strength and stiffness, lower production costs, renewability, and no problems with recycling, make it possible to effectively use them as fillers in the polymer matrix. It should be noted, however, that fibers of plant origin are characterized by a different chemical composition, including the share of cellulose, hemicellulose, and lignin, which directly affects their properties [19,20]. In order to improve the adhesion of fibers of plant origin to the polymer matrix, both physical and chemical methods of surface modification are used. One of them is the alkalization method. Fibers after alkalization are characterized by better adhesion to the polymer matrix, which directly improves the mechanical properties of such biocomposites. When analyzing the issue of using short fibers of plant origin, such aspects as the share of fibers in the polymer matrix, length of fibers, and their orientation [21,22,23,24,25,26] are considered. An important aspect of the production of composites with fillers of plant origin is the same method of their production. Single- and twin-screw extruders can be used for this. The best method cannot be directly indicated here because both positive and negative aspects that affect the properties of the obtained composites can be given for each [27]. In the case of the production of modern biocomposites with a PHBV matrix with a filler of plant origin, the extrusion process can be a problematic issue due to the sensitive, variable, and often difficult-to-predict processing conditions and properties of the processed biocomposites. In this work, an attempt was made to assess the impact of the selected extrusion method on the possibilities of production, processing, and an assessment of the properties of modern biocomposites with a PHBV matrix filled with flax and hemp fibers.

## 2. Materials and Methods

### 2.1. Materials

PHBV, under the trade name Enmat Y1000 of Helian Polymers (Belfeld, The Netherlands), in powder form, was used as the polymer matrix. Flax and hemp fibers produced by EKOTEX (Kowalowice, Poland) with a length of 1 mm were used as a filler in the polymer matrix. The mass fraction of fibers in the polymer matrix was 15 wt.%. Flax fibers were alkalized with 2% NaOH solution, while hemp fiber was alkalized with a 10% NaOH solution. The concentrations of the solutions were selected based on the results of the tests described in [26].

The biocomposites were produced on a twin-screw extruder (co-rotating method) and a single-screw extruder.

Due to the better presentation of the results, a set of markings used in the further part of the work was developed:

- H15TWIN—biocomposite with hemp fiber, produced using a twin-screw extruder;
- H15SINGLE—biocomposite with hemp fiber, produced using a single-screw extruder;
- F15TWIN—biocomposite with flax fiber, produced using a twin-screw extruder;
- F15SINGLE—biocomposite with flax fiber, produced using a single-screw extruder.

### 2.2. Composite Production and Sample Preparation

**Figure 1:**
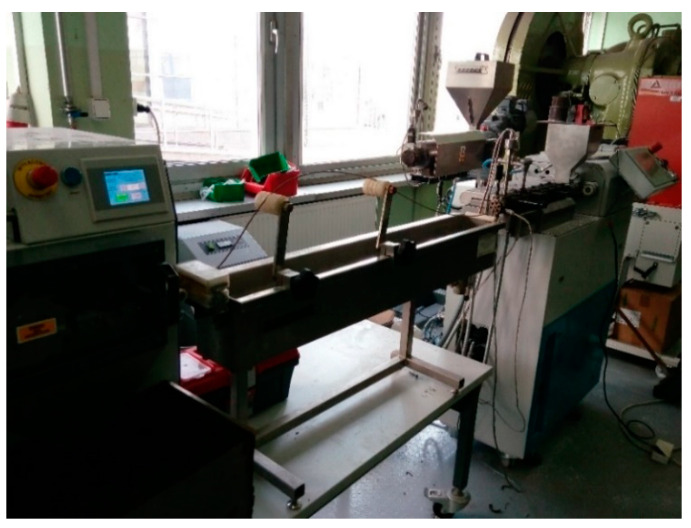
Extrusion line using ZAMAK REA-2P12A Explorer twin-screw extruder used to extrude the PHBV-hemp fiber and PHBV-flax-fiber biocomposites.

**Figure 2:**
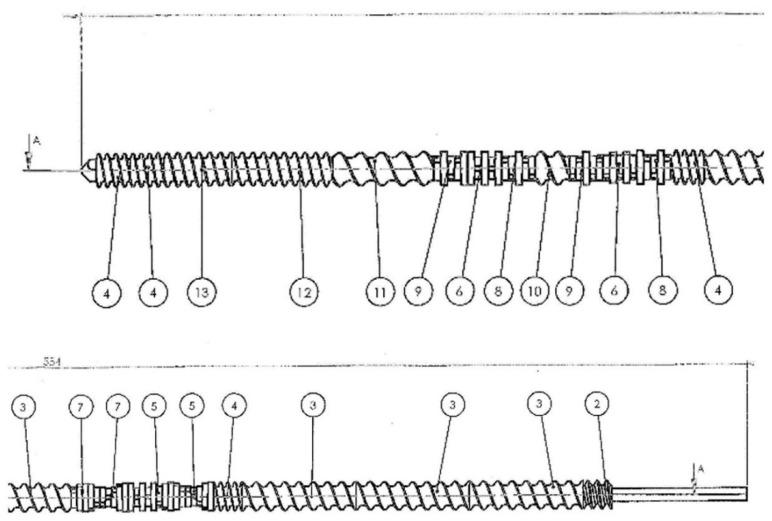
The geometry of the Ø12 screw of a twin-screw extruder with zones: 2—sealing segment; 3, 4, 10, 11, 12, 13—transport segments; 5, 6, 7, 8, 9—grinding segments (Source: [28]).

PHBV biocomposites filled with a fiber of plant origin were extruded using a ZAMAK REA-2P12A Explorer twin-screw extruder (produced by ZAMAK Mercator company, Skawina, Poland) (Figure 1). The technical data of the extruder are presented in Table 1. The construction of the screw of the twin-screw extruder with marks indicating the sealing, transport, and grinding zones is shown in Figure 2. Biocomposites were extruded by means of a twin-screw extruder at preset temperatures in the individual heating zones of the extruder (Table 2) at a constant screw speed of 50 rpm. Both the polymer matrix and plant fibers were dried before the extrusion process for 3 h at 90 °C.

**Figure 3:**
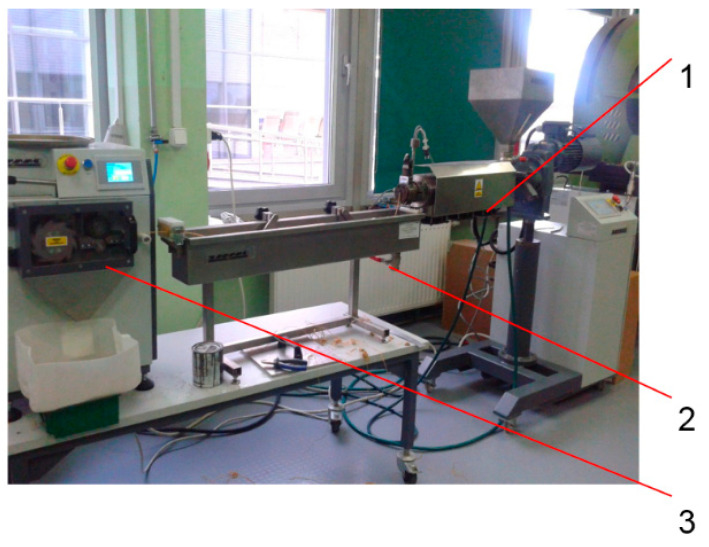
The extrusion line of PHBV- plant origin fiber biocomposites: 1—ZAMAK EHP-25E extruder, 2—cooling bath, 3—granulators.

**Figure 4:**
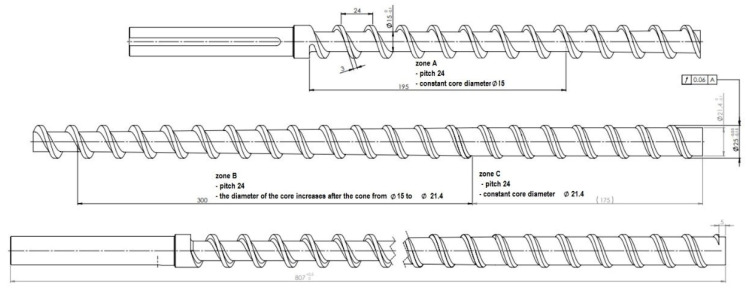
Geometry of a single-screw Ø25 extruder (Source: [29]).

PHBV biocomposites filled with the fiber of plant origin were also extruded using a ZAMAK EHP-25E single-screw extruder (produced by ZAMAK Mercator company, Skawina, Poland) (Figure 3). Technical data of the extruder are presented in Table 3. The construction of the screw of a single-screw extruder is shown in Figure 4. Both the polymer matrix and the fibers of plant origin were dried before the extrusion process for 3 h at 90 °C.

Initially, an attempt was made to extrude PHBV biocomposites with similar processing parameters as in the case of the twin-screw method. For the given similar values of temperatures of individual heating zones of the single-screw extruder, a high increase in extrudate viscosity was observed, whose consistency visually resembled a solid with a tendency to brittleness. Therefore, an attempt was made to optimize the processing parameters in order to obtain an extrudate with similar rheological parameters (viscosity and visual appearance) as produced using a twin-screw extruder. For this purpose, the temperature of the extruder head was increased to 170 °C (Table 4) and the rotational speed of the screw was set at 100 rpm. In the case of using a single-screw extruder, the extrusion efficiency was much higher due to the larger diameter of the screw and its higher rotational speeds.

### 2.3. Specimens Preparation

For the production of samples in the injection molding process, a DrBoy 55E injection molding machine (produced by BOY Maschines Inc., Exton, PA, USA) equipped with the Priamus system was used to monitor and control the injection molding process. An injection mold with inserts for uniaxial tensile testing (according to EN ISO 527-1) was used for the tests. All four types of biocomposites were injected with the same adjustable parameters presented in Table 5.

**Figure 5:**
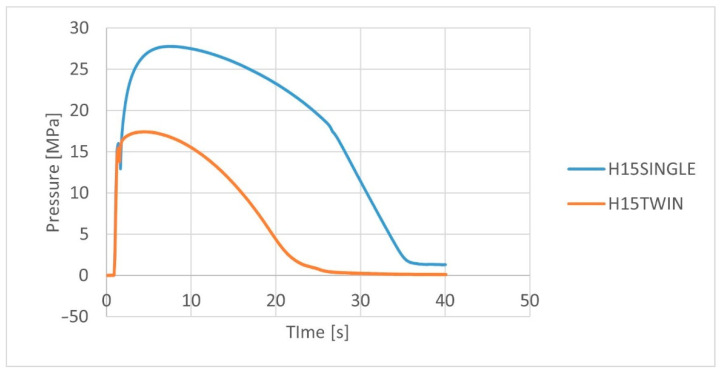
Variation of the cavity pressure for PHBV biocomposites with hemp fibers extruded by various methods.

**Figure 6:**
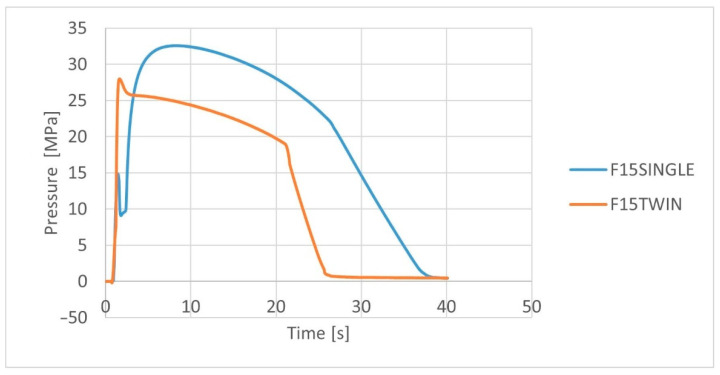
Variation of the cavity pressure for PHBV biocomposites with flax fibers extruded by various methods.

The analysis of the pressure profile in the cavity of the injection mold (Figure 5 and Figure 6) for biocomposites extruded by various methods allows the observation of higher pressure values for biocomposites extruded using a single-screw extruder. Already at this stage of the study results, a significant difference in the pressure values was observed, where the maximum pressure value for the H15TWIN and F15TWIN composites was approx. 17 MPa and 27 MPa, respectively, and for the H15SINGLE and F15SINGLE composites it was approx. 27 MPa and 33 MPa. As one can see, for both composites extruded by the twin-screw method, the maximum pressure values are lower than for composites extruded by the single-screw method.

### 2.4. Research Methods

A TA Instruments DSC Q2000 differential-scanning calorimeter (produced by TA Instruments, Inc., New Castle, DE, USA) was used for the thermal tests. All analyses were performed under a nitrogen atmosphere with a relatively constant flow of approximately 50 mL/min.

In order to assess the degradation temperature of the obtained biocomposites, TGA (thermogravimetric analysis) tests using the Mettler Toledo (Columbus, OH, USA) TGA device were carried out. In this method, the change in the mass of the tested sample with respect to the increasing temperature was determined. The samples were heated to 700 °C at a rate of 10 °C/min. Weight loss ranging from 1 to 10% was monitored.

The Zwick/Roell Z030 (produced by Zwick Roell, Ulm, Germany) uniaxial tensile testing machine was used to determine the strength properties of the obtained composites. The uniaxial tensile test was conducted in accordance with the EN ISO 527-1 standard for molded pieces with the dog-bone shape. Each series of samples consisted of seven pieces. Based on the obtained test results, the followings were determined: Young’s modulus (E), tensile strength (σ_M_), and relative elongation at maximum tensile stress (ε_M_). The results were evaluated through the statistical analysis. The arithmetic mean ($\bar{x}$), standard deviation (s), and coefficient of variation (V) were determined.

The hardness tests of biocomposites using the Brinell method were performed in accordance with the EN ISO 2039-1 standard in two areas of samples for uniaxial stretching, i.e., in the measuring zone for uniaxial tensile testing (zone A) and in the gripping part (zone B). A Zwick 3106 hardness tester (produced by Zwick Roell, Ulm, Germany) was used for this purpose. Seven samples were tested in each series.

Biocomposite samples were also tested in the impact tensile test. The impact tensile strength was determined in accordance with the PN-EN ISO 8256 standard. The CAEST 9050 (produced by Instron Inc. Europe, Buckinghamshire, UK) pendulum hammer was used for this purpose.

The morphology of the samples subject to tensile tests was examined using a HITACHI S-3400 scanning electron microscope (SEM) (produced by Hitachi Inc., Tokyo, Japan).

The degree of water absorption of the produced samples was tested on the basis of the EN ISO 62 standard. The shrinkage of the molded parts was carried out on the basis of EN ISO 294-4 standard. The primary shrinkage was tested after approximately 3 h, and the secondary shrinkage after approximately 14 days from the production of molded pieces in the injection molding process.

Preliminary fiber length measurements were also made using a Nikon LV-100D optical microscope (produced by Nikon Inc., Tokyo, Japan) and E-MAX software. The length and diameter of the fibers were measured on the surface layer of molded pieces for biocomposites extruded using the single- and twin-screw method. For each sample, the measurements were carried out in the same area, in the middle part of the dog-bone sample, intended for the uniaxial tensile test.

## 3. Results

When analyzing the results of tests carried out using differential scanning calorimetry (DSC) (Table 6), slightly higher values of glass transition, melting, and crystallization temperatures were found for biocomposites produced by the single-screw method. Taking into account the type of the filler, it was noticed that these temperatures for the biocomposites with flax fiber were slightly higher than for the composites with hemp fiber. However, it should be noted that these differences are within the statistical error. Markings were introduced in the table: specific heat—Δc_p_, glass transition temperature—T_g_, melting temperature—T_m_, and crystallization temperature—T_c_.

For biocomposites with a mass content of hemp fibers of 15 wt.% and produced by two different extrusion methods, thermal gravimetric analyses were performed. Higher degradation temperatures of biocomposites with flax fibers in relation to composites with hemp fibers were observed (Table 7). In turn, taking into account the extrusion method, higher degradation temperatures were found for both fillers in the case of biocomposites produced by the single-screw method.

**Figure 7:**
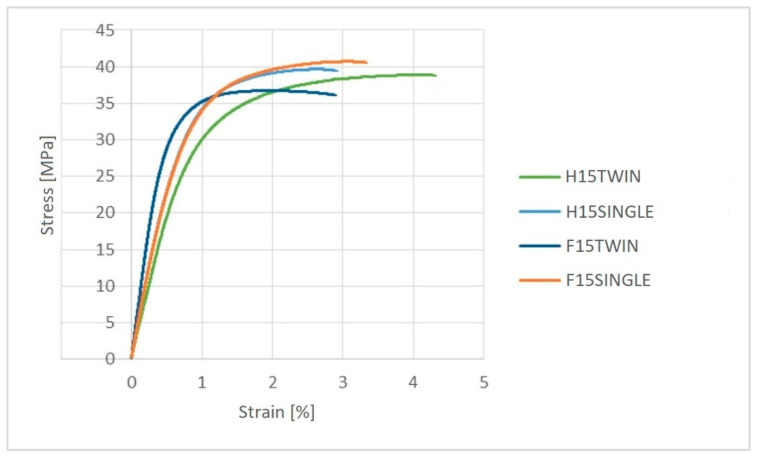
The stress–strain characteristics for PHBV biocomposites produced by single-screw and twin-screw extruders.

The obtained samples were also subject to a uniaxial tensile test. Exemplary stress-strain curves are presented in Figure 7. When analyzing the properties of biocomposites (Table 8) filled with hemp fiber produced in the single-screw extrusion process, an increase of approximately 31% in Young’s modulus and 2.5% in tensile strength and a decrease in elongation of 34%, relative to the biocomposite extruded on a twin-screw extruder, were observed. In the case of the biocomposite filled with the flax fibers, produced using a single-screw extruder, an increase of approximately 36% in Young’s modulus and 9% in tensile strength and a decrease in relative elongation at maximum tensile stress by the twin-screw extruder were observed.

**Figure 8:**
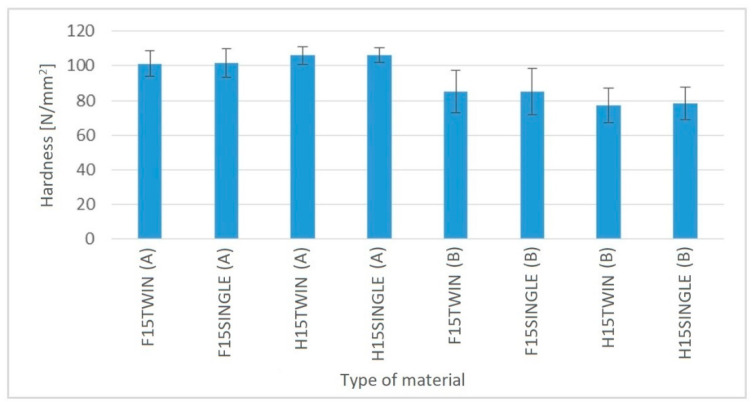
Hardness of PHBV biocomposites produced by single-screw and twin-screw extrusion.

In the case of hardness tests (Figure 8), no significant differences were found between biocomposites extruded using a single-screw and a twin-screw extruder. For the area A of the sample, i.e., the middle part of the dog-bone shaped sample intended for the uniaxial tensile test, the hardness values ranged from approx. 100 to approx. 107 N/mm^2^. In turn, in the area B, i.e., the gripping part of the dog-bone shaped sample, the hardness values ranged from 78 to 85 N/mm^2^.

**Figure 9:**
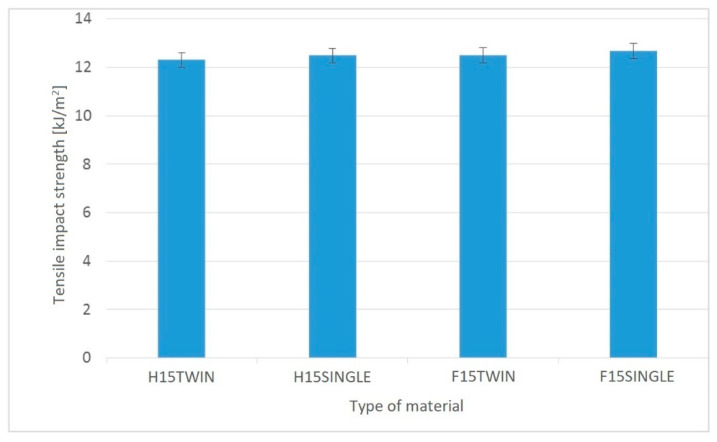
Impact tensile strength of PHBV biocomposites produced with single- and twin-screw extrusion.

When analyzing the results regarding the impact tensile strength (Figure 9), it can be seen that for PHBV biocomposites obtained using a single-screw extruder, the impact tensile strength is higher by approximately 1.5% compared to the same biocomposites produced using a twin-screw extruder. However, it should be noted that these results are within the statistical error range.

**Figure 10:**
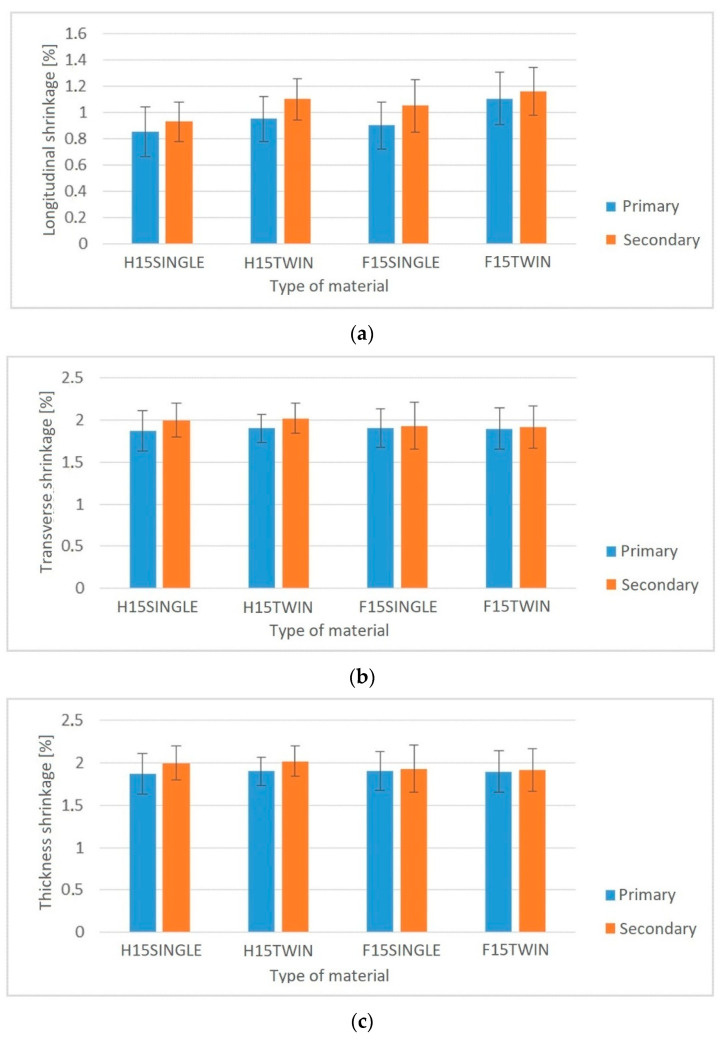
Primary and secondary shrinkage of injection moldings: (**a**) longitudinal shrinkage, (**b**) transverse shrinkage, (**c**) thickness shrinkage, for biocomposites produced by single- and twin-screw extrusion.

In the case of secondary shrinkage tests (Figure 10), lower longitudinal shrinkage values can be seen for biocomposites produced by single-screw extrusion (for the H1SINGLE composite, the shrinkage was lower and amounted to approximately 15%, while for F15SINGLE, the decrease in shrinkage was approximately 10%) compared to PHBV biocomposites produced using a twin-screw extruder. In the case of transverse shrinkage and thickness shrinkage, no significant changes were observed.

**Figure 11:**
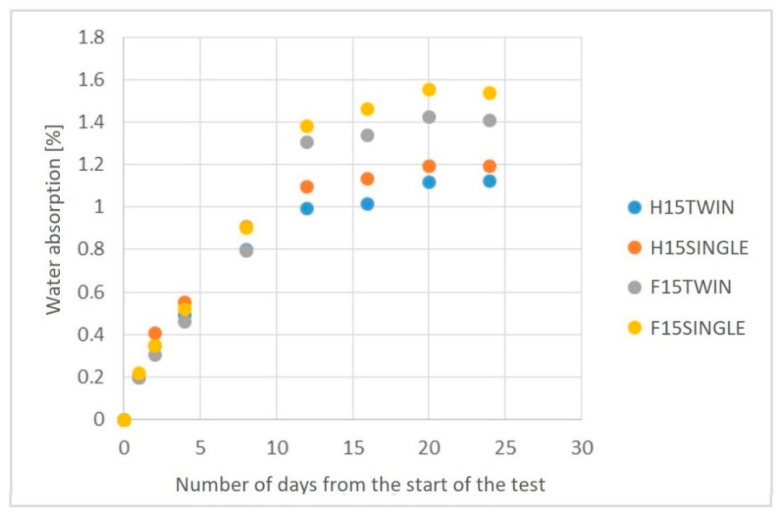
The water absorption of PHBV biocomposites produced by single- and twin-screw extrusion.

Based on the results regarding water absorption (Figure 11), a higher value of approximately 7% can be observed for PHBV biocomposites obtained by single-screw extrusion. The maximum water absorption after 24 days ranged from approximately 1.18% (for the H15TWIN biocomposite) to approximately 1.55% (for the F15SINGLE biocomposite). Based on the results obtained, it can be concluded that composites with flax fibers are characterized by greater water absorption than with hemp fibers.

**Figure 12:**
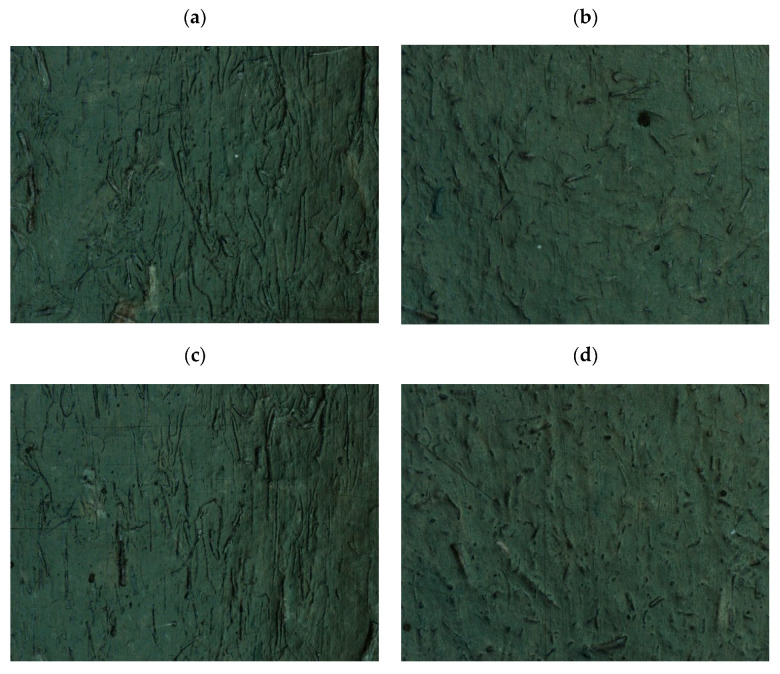
The photographs of fibers on the surface layer of PHBV biocomposite samples (magn. 50×): (**a**) H15SINGLE, (**b**) H15TWIN, (**c**) F15SINGLE, (**d**) F15TWIN.

Measurements of the fiber geometry on the surface layer of the compacts in Figure 12a,c indicate much longer fibers (for the single-screw method) compared to far fewer visible fibers on the surface of samples obtained from biocomposites produced on a twin-screw extruder (Figure 12b,d). The sample results of fiber geometry measurements, along with statistical analysis, are presented in Table 9.

**Figure 13:**
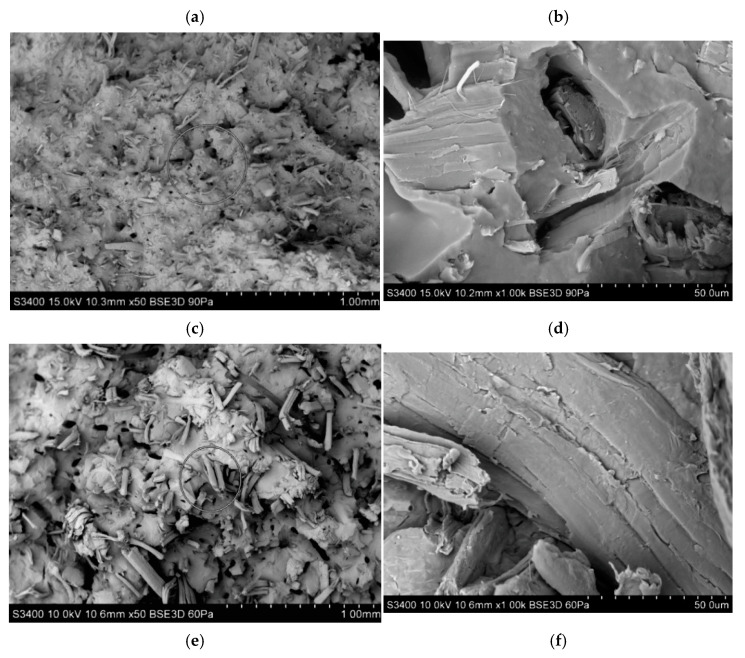

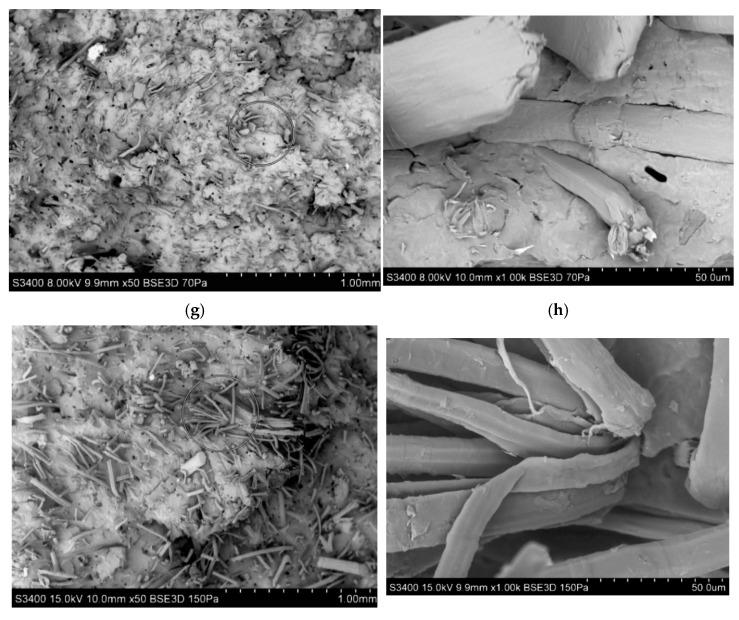
SEM photographs of fractures of PHBV composites: H15TWIN—(**a**) magn. 50×, (**b**) magn. 1000×, H15SINGLE—(**c**) magn. 50×, (**d**) magn. 1000×, F15TWIN—(**e**) magn. 50×, (**f**) magn. 1000×, F15SINGLE—(**g**) magn. 50×, (**h**) magn. 1000×.

By analyzing the SEM micrographs (Figure 13) of fractures of uniaxial stretched samples, a larger diameter and length of fibers were observed for PHBV biocomposites produced using a single-screw extruder. Their more developed surface, with visible dimples, was noted, which may indicate the greater porosity of the samples produced by single-screw extrusion.

## 4. Discussion

The extrusion of composites with fiber fillers consists of plasticizing the polymer, mixing it with fiber, and then transporting the mixture through the head to produce an extrudate. This process allows better homogenization of the material, which, if produced, (for instance) in the form of granules, will show much better homogeneity. As a consequence, this may result in the better quality of items produced by injection molding. The parameters that affect the properties of composites in the extrusion process include the rotational speed geometry of the screw/screws, number of heating zones, and temperature [30].

The rotational speed of the screw, when extruding composites, should be as high as possible to minimize the residence time of the material in the plasticizing unit, and to maximize the volumetric flow rate. However, when the extrudate is temperature sensitive, such as in cellulosic fibers, the speed of the extruder screw is limited by the maximum shear rate, which can degrade the fibers. Another reason for limiting the rotational speed of the screw is to minimize the degree of presence of air in the plasticizing unit. If the polymer is extruded at too-high speeds, the air may be “trapped”, which causes the formation of small air bubbles in the extrudate [31]. An important issue is also the assessment of the mechanical energy supplied to the fibers during the extrusion process. The appropriate amount of energy positively affects mixing and dispersion. The consequence of this is an improvement length-to-diameter ratio (conglomerates of fibers are stratified into fibers of smaller diameter), which increases the mechanical properties of the composite. Therefore, it is important to select such processing parameters (mainly the rotational speed of the screw) in order to avoid mechanical degradation of the fibers [30].

An important factor related to the temperature of the process is the residence time of the material in the plasticization unit, which may be too short to plasticize the material, or too long, which may lead to material degradation due to overheating [30,32]. In addition, the degradation of plant-derived fibers can occur in composites for which the melting point of the polymer is higher than the fiber degradation temperature. In the paper [33] by Summerscales and co-authors, the temperature of 200 °C was indicated as the temperature of cellulose degradation in the fibers. In addition, the authors noticed a direct relationship between the time of heating of the fibers and the temperature, which directly affects the degradation and, consequently, the deterioration of the mechanical properties of the fibers. Similar conclusions were obtained by Van de Velde and Kiekens [34,35], indicating that thermal treatment of fibers at 180 °C for 15 min resulted in the degradation of flax fibers. Wielage et al. [36] studied the thermal stability of flax and hemp fibers using DSC and TGA. A slight decrease in the mass of natural fibers was observed in the temperature range of 200–220 °C. Above 220 °C, irreversible degradation of the fibers was observed. It was found that the temperature influenced the color of the obtained extrudate, therefore it also determined the aesthetic values of the products.

Another important issue is the geometry of the screw in the plasticizing unit of extruders, which varies in individual zones. These zones have different purposes (e.g., mixing, grinding, shearing, or transferring). Shearing can be increased by using mixing and crushing elements [37]. In the work of Lertwimolnun and co-authors [38], it was shown that the construction of the screw had a significant impact on delamination of the organoclay in the PP matrix. Moreover, it was noted that a too-long extrusion profile negatively affected the course of the process.

The extrusion with the use of single- and twin-screw extruders provides a different course of shearing and homogenization of the processed material during the extrusion process [39]. Basically, twin-screw extruders are used to extrude heterogeneous materials, i.e., composites with fibers or nanoadditives. The advantage of the process using the above-mentioned machines is the uniform distribution of inclusions in the polymer matrix in relation to the single-screw extruder [40]. In the case of using fibrous fillers of natural origin, a major problem may be the shortening and degradation of fibers as a result of high stress values, despite correctly selected process parameters. In turn, it should be noted that the use of a single-screw extruder may result in a reduction in the shear rate in order to maintain the appropriate degree of homogenization [41,42].

The results of tests produced by single- and twin-screw extrusion of PHBV biocomposites indicate a significant impact of the extrusion method used on their properties. Biocomposites produced using a single-screw extruder were characterized by better mechanical properties in the uniaxial tensile test. This may be due to the fact that the fibers did not undergo mechanical degradation (shortening) during extrusion in this method (compared to biocomposites produced by twin-screw extrusion). The biocomposite produced by single-screw extrusion spent less time in the plasticizing system due to the standard profile of the screw, the larger diameter of the cylinder channel and the screw, as well as the higher rotational speed of the screw. Worse mechanical properties of biocomposites produced by twin-screw extrusion could result from the small diameter of the screw, the presence of grinding zones in the screw profile (which could result in shortening of the fibers), and the large ratio of the screw length to its diameter (which could cause thermal degradation of the biocomposite). It should be mentioned, however, that the biocomposites produced using a twin-screw extruder were characterized by lower porosity and a lower degree of water absorption, which may be due to the fact that the twin-screw extruder was equipped with a degassing module for the plasticized biocomposite during the extrusion process.

The phenomenon of fiber shortening may be confirmed by, for example, the observed changes in processing shrinkage in the length direction of the compact. Both for biocomposites with the flax and hemp fibers produced by the single-screw method, the shrinkage along the length was lower than for biocomposites produced by the single-screw method. The fibers act as a kind of reinforcement, where when they are arranged in the longitudinal direction, they limit shrinkage in this direction. Shorter fibers of smaller length and diameter limit this type of shrinkage to a lesser extent, which was observed for biocomposites produced by the twin-screw method.

Measurements of the fiber geometry on the surface layer of the molded piece carried out for the composite obtained using the single-screw method indicate the presence of much longer fibers compared to the fibers on the surface of samples obtained from biocomposites produced on a twin-screw extruder, which are less visible. It seems that this may be due to the fact that the fibers in the composite obtained in the single-screw extrusion technology can be much thicker, stiffer, and longer. Due to this fact, they also become more visible on the surface layer. In addition, a reduction in the thickness and length of fibers embedded in a polymer matrix for biocomposites produced by the twin-screw method was also observed, as confirmed by SEM images, also with regard to SEM images of fibers not embedded in the polymer matrix [26].

The observed phenomenon of fiber shortening in the extrusion process was also noted by other researchers. In the work of Baiardo et al. [43], an extrusion process was carried out for a polyester composite with the flax fiber as a filler. The experiment was carried out using a rotor mixer under the following conditions: process temperature 120 °C, rotational speed of the screw in the range of 10 to 50 rpm, and extrusion time of 5 to 15 min. The length of the fibers ranged from 0.25 to 0.9 mm. It was noticed that the longer the extrusion time and the higher rotational speed of the screw, the fibers were shortened, which resulted in a decrease in the strength properties of the composite. On the other hand, in the work of Berzin and co-authors [44] the extrusion was carried out using a twin-screw extruder of the polycarbonate-hemp fiber composite. At a temperature of 100 °C, the extrusion of the composite was carried out at a variable rotational speed of screw in the range of 100 to 300 rpm. A shortening of the length of the fibers was observed with the increase of the screw rotational speed. On the other hand, El-Sabbagh and co-authors [45] proved in their research for a PP-flax fiber composite extruded using a twin-screw extruder that the diameter of the fibers was reduced by 10% and the fibers were shortened, depending on the rotational speed of the screw.

When choosing the right method of extrusion of PHBV-flax fiber biocomposites, the aspect of process parameters and efficiency should also be analyzed. In the case of the production of composites using a single-screw extruder, the temperature of individual heating zones of the extruder had to be increased in order to obtain an extrudate with a similar viscosity as in the case of production using a twin-screw extruder. In the case of using a single-screw extruder, it was possible to set higher rotational speeds, thanks to which the extrusion efficiency was significantly higher. In addition, the availability of single-screw extruders in manufacturing companies is much greater than in the case of twin-screw equipment. On the other hand, the most alarming aspect was that the homogenization of the fiber-matrix phases did not differ significantly in relation to both extrusion methods. In the case of fibers after the twin-screw method, they are shorter, which increases their number in the matrix, hence SEM images can show a greater number of them in the polymer matrix, and thus a denser arrangement. In the case of SEM images for composites produced by the single-screw method, the fibers are larger (they have not been shortened), and thus they are less often embedded in the polymer matrix. By analyzing the results of all tests carried out in statistical terms, it was found that the standard deviation for test samples of individual composites differs slightly with a slight advantage of the twin-screw method, which is an obvious phenomenon. If in the case of any of the methods the reproducibility of the results decreased (the dispersion would be greater), then our assumptions would be directed to the low homogenization of the fiber-matrix phases due to the uneven distribution of fibers in the polymer matrix.

The consequences of using a given extrusion method in relation to the subsequent history of composites processing should also be analyzed. In the injection process, higher values of pressure in the molding cavity were noted for biocomposites produced using a single-screw extruder, which may indicate higher flow resistance for biocomposites with fibers that were less shortened or stratified during single-screw extrusion. In this approach, the biocomposite produced by the twin-screw method with fibers of a smaller diameter and length is easier to process in the context of the possibility of filling the mold cavity.

Based on the results of the TGA tests, higher degradation temperatures were found in relation to the percentage weight loss for biocomposites produced by the single-screw method. On the other hand, on the basis of DSC studies, slightly higher values of glass transition, melting, and crystallization temperatures were noted for biocomposites produced by the single-screw method. This may be related to the smaller heating surface of the fibers with a greater length and diameter in relation to the chipped fibers of biocomposites produced by the twin-screw method. In addition, changes in the properties of composites may also be the result of polymer degradation, which would explain the changes in transition and melting temperature and the results obtained with TGA. The heating time of the fibers should also be mentioned. In the work of Summerscales et al. [33], it was noted that the heating time of the fibers directly affected their degradation, reducing the mechanical properties of composites. The length-to-diameter ratio of the screws in the twin-screw extruder (Table 1) was much greater than in the single-screw extruder (Table 3), which resulted in a longer stay of the biocomposite in the plasticization unit and could also contribute to the thermal degradation of the fibers.

## 5. Conclusions

A comparative analysis concerning the selection of the extrusion method for biocomposites with a PHBV matrix filled with flax and hemp fibers was performed in this study. In the manufacturing of biocomposites it was not possible to select the same setting parameters of the process due to the different microstructure forms of the obtained extrudate. In order to unify the form of the obtained biocomposites, it was necessary to increase the temperature and speed of extrusion in the single-screw method. This resulted in an increase in extrusion efficiency.

In the context of assessing the mechanical properties and quality of the moldings, the deterioration of properties in the uniaxial tensile test and the increase in shrinkage of molded piece for biocomposites produced by the twin-screw method were noted. The reason for this phenomenon is most likely the fact that the fibers during processing with this method were shortened and stratified, and could undergo thermal degradation due to the specific arrangement of the screw zones (transport, grinding, sealing) and the too-long stay in the plasticization system due to the very high length-to-diameter ratio of the screw.

Despite the indicated disadvantages of the twin-screw method, some advantages for the processing of the analyzed biocomposites can be indicated. The fibers are more fragmented, hence lower pressure values in the injection mold were obtained due to lower flow resistance. This allows the easier shaping of products in the injection molding process. In addition, biocomposites produced using a twin-screw extruder were characterized by lower porosity and a lower degree of water absorption, which could be the result of the use of a degassing zone in the twin-screw extruder.

Summing up, it is difficult to unequivocally recommend the method of extrusion for biocomposites with PHBV matrix filled with flax and hemp fibers. However, when analyzing the issue of commercialization of this type of biocomposites, the availability of single-screw extruders is greater. The obtained properties of the injection products indicate the choice of single-screw extrusion. The products can be then characterized by slightly better mechanical properties and lower processing shrinkage.

## Figures and Tables

**Table 1:** Technical data of the ZAMAK REA-2P12A Explorer extruder (based on [28]).

**Screw**	
Nominal diameter [mm]	Ø12
Working length [mm]	480
The ratio of length to the diameter	40
Nominal rotational speed of the (stepless adjustable) [rpm]	800
**Cylinder**	
Maximum pressure [MPa]	10
Heater heating power [W]	900
Temperature adjustment range [°C]	10–400
Number of heating and cooling zones	7
Cooling method	air

**Table 2:** The set temperature of the heating zones for the twin-screw extruder.

Head	Zone 7	Zone 6	Zone 5	Zone 4	Zone 3	Zone 2	Zone 1	Charge
160 °C	160 °C	160 °C	160 °C	160 °C	160 °C	155 °C	145 °C	50 °C

**Table 3:** Technical data of the ZAMAK EHP-25E extruder (based on [29]).

**Screw**	
Nominal diameter [mm]	Ø25
Working length [mm]	670
Length to diameter ratio	26.8
Nominal rotational speed (stepless adjustable) [rpm]	200
**Cylinder**	
Maximum pressure [MPa]	20
Heater heating power [W]	1500
Temperature adjustment range [°C]	30–400
Number of heating and cooling zones	3
Cooling method	air

**Table 4:** Temperature of heating zones for a single-screw extruder.

Head	Zone 3	Zone 2	Zone 1	Charge
170 °C	165 °C	155 °C	145 °C	35 °C

**Table 5:** The processing parameters of samples for uniaxial tensile testing.

Parameter	Value
Melt temperature [°C]	167
Cooling time [s]	25
Packing time [s]	25
Packing pressure [MPa]	30
Injection rate [cm^3^/s]	35
Mold temperature [°C]	60

**Table 6:** Selected thermal properties of PHBV biocomposites containing 15% (by weight) of hemp or flax fibers produced in the extrusion technology.

Biocomposite Type	Δc_p_ [J·g^−1^·°C^−1^]	T_g_ [°C]	T_m_ [°C]	T_c_ [°C]
H15TWIN	0.055	5.40	162.90	97.80
H15SINGLE	0.072	7.30	163.85	100.0
F15TWIN	0.077	6.40	164.30	101.10
F15SINGLE	0.079	7.50	166.65	104.30

**Table 7:** Composite weight loss as a function of temperature for PHBV biocomposites with 15 wt.% share of hemp and flax fibers using different extrusion methods.

Loss of Weight [%]	Temperature Change for Biocomposites Type [°C]			
	H15TWIN	H15SINGLE	F15TWIN	F15SINGLE
1	87.61	91.91	96.88	100.71
2	258.26	263.17	247.8	252.68
3	267.21	270.01	257.68	270.41
4	270.57	273.06	262.69	273.07
5	272.56	274.99	265.82	273.69
6	273.97	276.39	267.96	278.54
7	275.05	277.47	269.58	282.23
8	275.93	278.36	270.88	286.94
9	276.66	279.06	271.95	288.46
10	277.30	279.68	272.84	289.12

**Table 8:** The results of the static tensile test for biocomposites produced using single-screw and twin-screw extruders.

Type of Material	Statistics	E [MPa]	σ_M_ [MPa]	ε_M_ [%]
H15TWIN	$\bar{x}$	3992.55	38.11	4.05
	s	43.10	1.08	0.14
	V	1.12	2.84	3.45
H15SINGLE	$\bar{x}$	5242.29	39.03	2.68
	s	196.29	0.59	0.17
	V	3.74	1.51	6.27
F15TWIN	$\bar{x}$	3860.63	36.94	4.21
	s	45.22	0.34	0.12
	V	1.17	0.98	3.38
F15SINGLE	$\bar{x}$	5257.86	40.16	3.08
	s	131.23	0.38	0.21
	V	2.50	0.94	6.72

**Table 9:** The results of fiber geometry measured on the top layer of the sample.

Biocomposite	L—Length [mm]			D—Diameter [mm]			Aspect Ratio L/d
	$\bar{x}$	s	V	$\bar{x}$	s	V	
H15SINGLE	1.125	0.163	14.489	0.123	0.029	23.577	9.146
H15TWIN	0.793	0.231	29.130	0.095	0.043	45.474	8.347
F15SINGLE	1.051	0.179	17.031	0.113	0.032	28.319	9.301
F15TWIN	0.817	0.282	34.517	0.093	0.041	44.301	8.785

## Data Availability

The data presented in this study are available on request from the corresponding author.
